# Concomitant downregulation of the imprinted genes *DLK1* and *MEG3* at 14q32.2 by epigenetic mechanisms in urothelial carcinoma

**DOI:** 10.1186/1868-7083-6-29

**Published:** 2014-11-23

**Authors:** Annemarie Greife, Judith Knievel, Teodora Ribarska, Günter Niegisch, Wolfgang A Schulz

**Affiliations:** Department of Urology, Medical Faculty, Heinrich-Heine University, Moorenstr. 5, Düsseldorf, 40225 Germany

**Keywords:** *DLK1*, *MEG3*, Urothelial cancer, Imprinted genes, DNA methylation, Histone modification

## Abstract

**Background:**

The two oppositely imprinted and expressed genes, *DLK1* and *MEG3*, are located in the same gene cluster at 14q32. Previous studies in bladder cancer have suggested that tumor suppressor genes are located in this region, but these have not been identified.

**Results:**

We observed that both *DLK1* and *MEG3* are frequently silenced in urothelial cancer tissues and cell lines. The concomitant downregulation of the two genes is difficult to explain by known mechanisms for inactivating imprinted genes, namely deletion of active alleles or epitype switching. Indeed, quantitative PCR revealed more frequent copy number gains than losses in the gene cluster that were, moreover, consistent within each sample, excluding gene losses as the cause of downregulation. Instead, we observed distinctive epigenetic alterations at the three regions controlling *DLK1* and *MEG3* expression, namely the *DLK1* promoter; the intergenic (IG) and *MEG3* differentially methylated regions (DMRs). Bisulfite sequencing and pyrosequencing revealed novel patterns of DNA methylation in tumor cells, which were distinct from that of either paternal allele. Furthermore, chromatin immunoprecipitation demonstrated loss of active and gain of repressive histone modifications at all regulatory sequences.

**Conclusions:**

Our data support the idea that the main cause of the prevalent downregulation of *DLK1* and *MEG3* in urothelial carcinoma is epigenetic silencing across the 14q32 imprinted gene cluster, resulting in the unusual concomitant inactivation of oppositely expressed and imprinted genes.

**Electronic supplementary material:**

The online version of this article (doi:10.1186/1868-7083-6-29) contains supplementary material, which is available to authorized users.

## Background

The differential expression of alleles inherited from mother or father at genomic imprinted genes is achieved by epigenetic mechanisms, particularly by differential methylation at regulatory regions designated as differentially methylated regions (DMRs). Imprinted genes regulate growth and other physiological functions during embryonic development, but also in adult tissues. Since several maternally imprinted genes limit growth, they possess tumor-suppressive potential and tend to become inactivated in different types of human cancer [1]. Their inactivation in cancers is brought about by deletion of the active allele or by a change of the epigenetic state of the active allele to that of the inactive one, that is, epitype switching. Importantly, either mechanism results in a homogeneous epigenetic state that corresponds to that of the normally inactive paternal allele. A well-studied example is the imprinted tumor suppressor gene *CDKN1C*, which is inactivated alternatively by genetic or epigenetic mechanisms in several human cancers, including urothelial carcinoma [2, 3].

**Figure 1:**
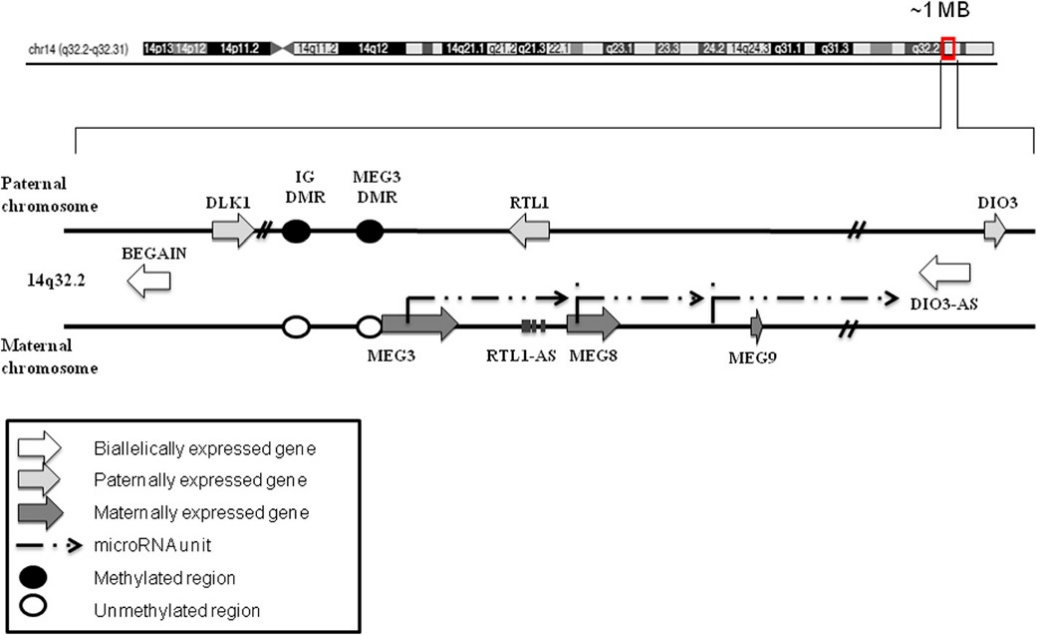
**Schematic presentation of the** ***DLK1-DIO3*** **imprinting cluster at chromosome 14q32.2** ***.***The *DLK1-DIO3* cluster contains three paternally expressed protein coding genes (light gray arrows) and multiple maternally expressed noncoding RNAs (dark gray arrows). The respective inactive gene copies are not shown. It is debated whether *BEGAIN* and *DIO3-AS* (white) are biallelically expressed. Arrowheads indicate the direction of transcription. Imprinting is regulated by differentially methylated regions (DMR), the IG DMR and the *MEG3* DMR, methylated at the paternal allele (black circle) and unmethylated at the maternal allele (white circle). The relative localizations of selected microRNAs and the C/D RNA unit are indicated by dashed arrowheads.

In several cancers, a cluster of imprinted genes at 14q32.2, the *DLK1-MEG3* cluster, is affected by allelic losses or epigenetic changes [4–7]. This cluster comprises several protein-coding and nonprotein-coding genes (ncRNAs), including antisense RNAs (asRNAs), small nucleolar RNAs (snoRNAs or C/D RNAs) and microRNAs (miRNAs) (Figure 1). The paternally expressed genes include the three protein-coding genes *Delta-like 1* (*DLK1), Deiodinase Iodothyronine Type III* (*DIO3*) and *Retrotransposon-like Gene 1* (*RTL1* or *PEG11*) [8]. The maternally expressed genes *Maternally Expressed Gene 3* (*MEG3*), *Maternally Expressed Gene 8* (*MEG8*) and *RTL1 antisense (RTL1-AS)* [9, 10] encode long noncoding RNAs. Gene expression in the cluster is controlled by differentially methylated regions (DMR) located 11 kb upstream of *MEG3* (intergenic differentially methylated region, IG DMR) and 1.3 kb upstream of the *MEG3* transcription start site *(MEG3* DMR*)* [11]. DNA methylation in the *DLK1* promoter is also relevant for its expression. The IG DMR*,* which is methylated on the paternal allele and unmethylated on the maternal allele, serves as the initial imprinting control region (ICR) for the entire cluster during early development [12], whereas in adult tissues the *MEG3* DMR usually represents the dominant regulatory region [13]. The expression of *DLK1* and *MEG3* is commonly reciprocal, possibly as a consequence of regulatory effects of the *MEG3* RNA [12].

Loss of imprinting in the 14q32 region due to epimutations at the IG DMR or microdeletions has been implicated in a range of diseases including UPD14mat/pat (uniparental disomy 14) and various cancers [4–7]. In renal and hepatic cancers and certain leukemia, a loss of *DLK1* expression is associated with changes in DNA methylation at this gene and its control regions [5, 14–16]. *MEG3* has been reported to act as a tumor suppressor in a broader range of cancers [1, 17–19]. Both DLK1 and *MEG3* exert various functions relevant for cancer development and progression, including regulation of growth factors and Notch signaling by DLK1, and regulation of TP53, pRB1 and NOTCH activity by *MEG3* [20–22].

Urothelial carcinoma is the most common cancer of the urinary bladder. It can be categorized into two subtypes, namely papillary tumors and the more malignant invasive carcinomas, which are characterized by pronounced chromosomal instability [23, 24]. In particular, more than 30% of invasive urothelial cancers, especially high stage cases, have been reported to contain losses at 14q32 [25–28]. It is therefore thought that the region harbors a tumor suppressor gene antagonizing cancer progression. Given their known functions and the findings in other cancers, *DLK1* and *MEG3* are good tumor-suppressor candidates. Indeed, *MEG3* has recently been reported to become downregulated in the majority of urothelial carcinomas and to exert tumor-suppressive functions [29]. However, the mechanism of its downregulation has not been investigated, yet.

Unexpectedly, we found that expression of both *DLK1* and *MEG3* was strongly diminished in urothelial carcinoma tissues and cell lines. This finding raises a conundrum as it is difficult to envision how either allelic loss or epitype switching could lead to the concomitant downregulation of these two imprinted and normally inversely expressed genes, which are located less than 100 kb apart. Indeed, upon closer investigation, we found that inactivation of the two genes is associated with the establishment of a novel epigenetic state in the region, which is distinct from that of either parental allele and is independent of copy number changes in most urothelial carcinoma tissues and cell lines. This epigenetic state involves a characteristic DNA methylation pattern and a strong shift towards repressive histone modifications across three major regulatory regions in this imprinted gene cluster. This mechanism could provide a means to silence both genes despite their normal opposite regulation.

## Results

### *DLK1*and *MEG3*expression are concomitantly diminished in urothelial cancer

**Figure 2:**
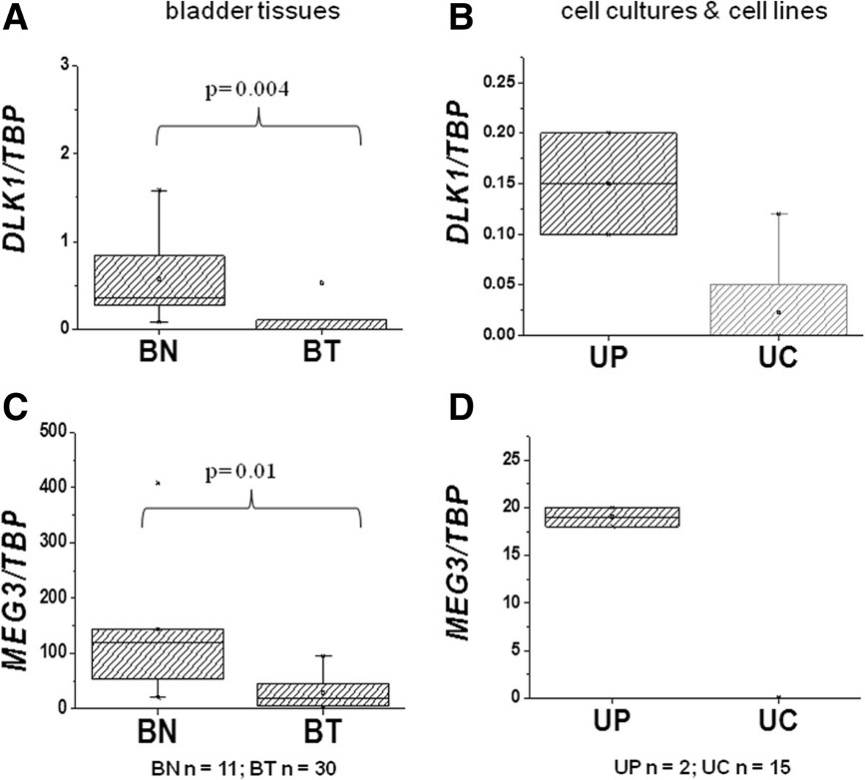
**DLK1 and MEG3 expression in urothelial tissues and cells.** Quantitative reverse polymerase chain reaction (RT-PCR) analysis of gene expression relative to the reference gene *TBP.* **(A, C)** Boxplot representations of *DLK1* (*P* = 0.004) and *MEG3* expression (*P* = 0.01) in 11 benign (BN) versus 30 cancerous (BT) urothelial tissue samples. **(B, D)** *DLK1* and *MEG3* expression in cultured normal urothelial cells (UP) and urothelial carcinoma cell lines (papillary: BC61, BFTC905, J82, RT4, RT112, SW1710; invasive: 5637, 639v, 647v, HT1376, SD, T24, VmCub1, Umuc3) in comparison to HepG2 hepatoma cells. Statistical comparisons between benign bladder and tumor bladder tissue expression were made by the Mann-Whitney U Test with SPSS 21.

Initially, we quantified *DLK1* and *MEG3* mRNA by qPCR in urothelial cancer tissues (n = 30) and cell lines compared to benign bladder tissue samples (n = 11) and primary cultured normal urothelial cells. Normal kidney tissue and the hepatoma cell line HepG2 were used as additional positive controls. *DLK1* mRNA was significantly reduced in urothelial cancer tissues compared to normal bladder tissue (Figure 2A) and was low or undetectable in all investigated urothelial carcinoma cell lines but remained detectable, albeit at lower levels, in cultured normal urothelial cells (Figure 2B). *MEG3* was robustly expressed in benign bladder tissues and more moderately in normal urothelial cells, but was significantly reduced in urothelial cancer tissues and totally absent in urothelial cancer cell lines (Figure 2C and D). In accord with previous reports [5, 30] HepG2 expressed only *DLK1*.

### Downregulation of *DLK1*and *MEG3*occurs irrespective of frequent copy number gains and losses at 14q32.2

**Figure 3:**
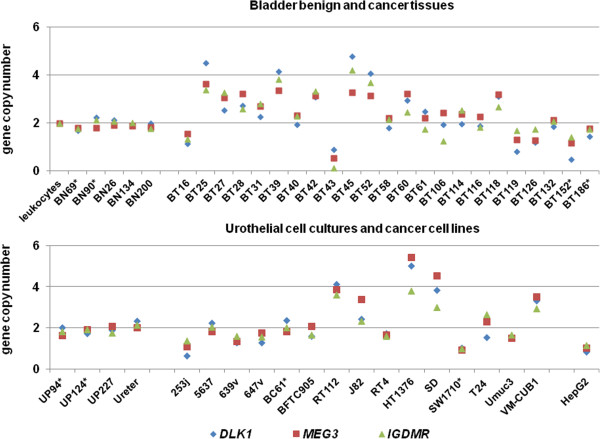
**Gene copy number analysis of the 14q32 imprinting cluster.** Gene copy numbers analysis by qPCR of the *DLK1* promoter (blue squares), the IG DMR (green rhombi) and the *MEG3 DMR* (red triangles) in leukocytes, benign (BN) and cancerous (BT) bladder tissues, urothelial cancer cell lines and normal urothelial cells (UP). Copy numbers were normalized to those in leukocytes set as 2 and to *GAPDH* as a reference gene. The asterisks indicate samples analyzed for DNA methylation by bisulfite sequencing. All other samples were analyzed only by pyrosequencing.

We measured copy number changes at *DLK1* and both DMRs in the chromosomal region 14q32.2 by qPCR in bladder cancer tissues and cell lines to assess whether gene deletions were responsible for the decreased expression of the two genes in urothelial carcinoma (Figure 3).

In benign bladder tissue samples (BN) measured copy numbers varied between 1.7 and 2.2, as normalized to normal diploid leukocytes set at two copies. Of 23 urothelial carcinoma samples (BT), 10 cases displayed increased copy numbers and 5 cases had decreased copy numbers, whereas 8 tumors showed copy numbers in the normal range (1.7 to 2.2). Importantly, gene copy number changes affected all three analyzed loci to the same extent within each sample.

Similarly, gene copy number changes affected all analyzed genes to the same extent in urothelial cancer cell lines (UC). Primary urothelial cell cultures (UP) were measured as diploid, as expected. Seven cell lines displayed elevated copy numbers between 2.5 and 3.5 across the analyzed region, for example, the Umuc3 and 639v cell lines. In accord with our results the predicted modal copy numbers are 3 for Umuc3 and 639v [31]. Decreased copy numbers were measured in two urothelial cancer cell lines and in HepG2. The decreased copy number measured for SW1710 is compatible with its hypotetraploid karyotype with one or two chromosomes number 14 per cell. Six cancer cell lines retained normal copy numbers. As in tumor tissues, all copy numbers were very similar, in the range of the technical variation (< ±10%), for the three loci.

Thus, whereas copy numbers of the 14q region were variously increased or decreased in urothelial carcinoma tissues and cell lines, *MEG3* and *DLK1* expression was reduced in almost all urothelial cancer tissues and cell lines, irrespective of copy numbers [see Additional file 1: Table S2].

### Urothelial cancers display distinctive DNA methylation changes in the chromosome 14q32.2 imprinted gene cluster

To investigate whether *MEG3* and *DLK1* downregulation was associated with changes in DNA methylation, we analyzed the three relevant CpG-rich regulatory regions, the *DLK1* promoter, the IG DMR, and the *MEG3* DMR*,* by bisulfite sequencing in selected urothelial cancer tissue and cell line samples (marked by asterisks in Figure 3). The analysis included two urothelial cancer cells lines representing extremes in differentiation and invasiveness (highly invasive SW1710 versus well-differentiated BC61 cells) and HepG2 as a control for a *DLK1* expressing cell line.

**Figure 4:**
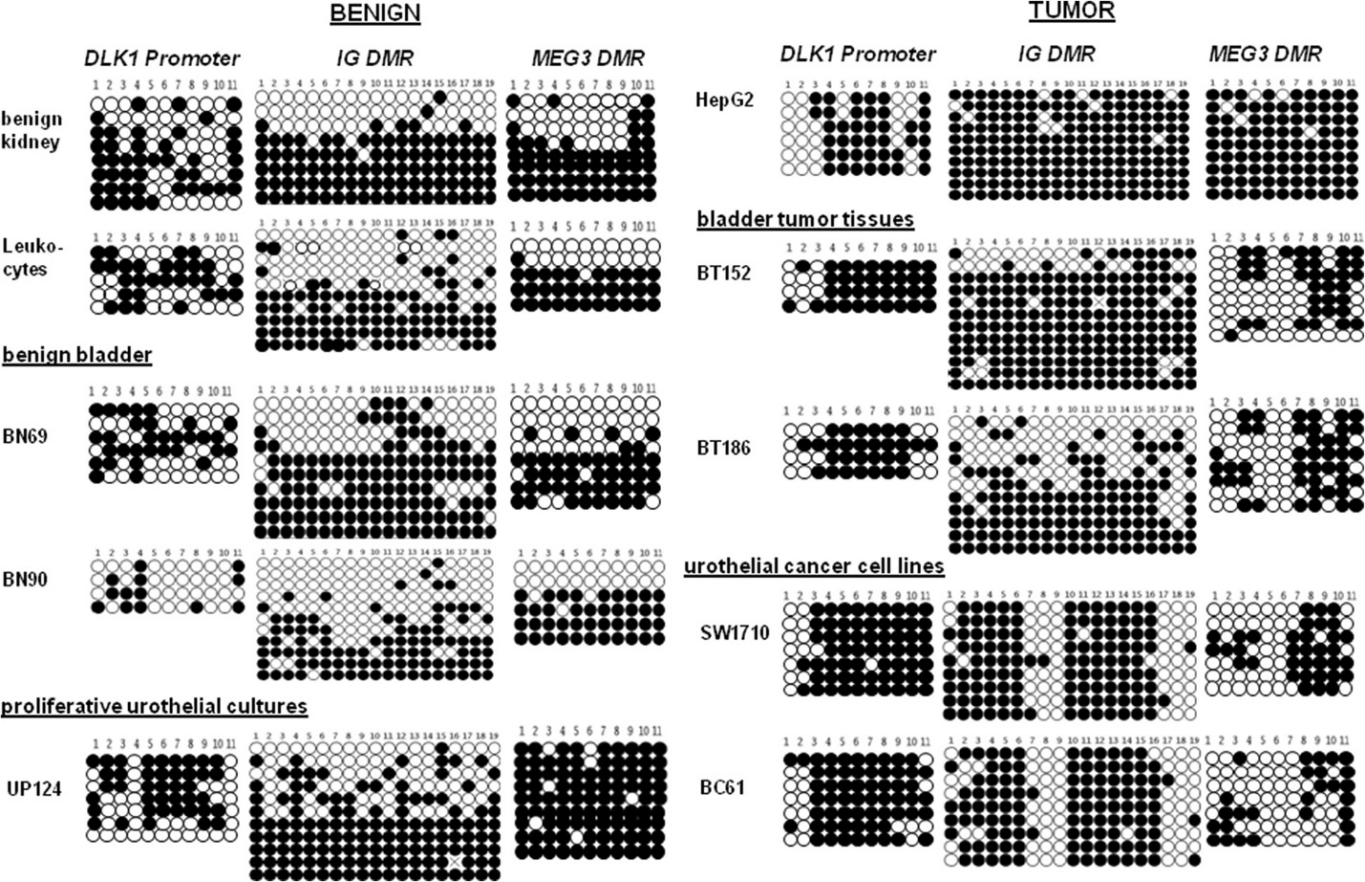
**DNA methylation analysis of control regions in the 14q32 imprinted gene cluster.** Bisulfite sequencing results of 11 CpGs in the *DLK1* promoter region (UCSC gene position 101192721-101192924, UCSC genome browser version 2009, Hg19), 19 CpGs in the IG DMR (UCSC gene position 101277184-101277612) and the *MEG3 DMR* (UCSC gene position 101290923-101291134) in benign (left side) and tumor (right side) samples. As benign samples, a normal kidney, leukocytes, two bladder tissues (BN) and primary cultured urothelial cells were analyzed. Tumor samples were urothelial cancer tissues (BT), the bladder cancer cell lines SW1710 and BC61, as well as the hepatoma cell line HepG2. Black circles indicate methylated CpGs, whereas white circles indicate unmethylated CpGs.

The *DLK1* promoter was partly methylated in benign kidney and bladder tissues as well as in leukocytes (Figure 4, left). While the methylation of individual alleles was heterogeneous, there was a tendency for the more distal CpGs (1-4) to be methylated and the central CpGs (5-10) to be unmethylated. In contrast, in urothelial cancer cell lines (SW1710, BC61) and urothelial cancer tissues (BT152 and BT186), methylation at the promoter assumed a homogeneous pattern with methylated CpGs at the center of the sequence flanked by unmethylated CpG sites at its margins (Figure 4). A tendency towards this pattern was also seen in HepG2 (Figure 4).

Bisulfite sequencing analysis of the IG DMR in normal kidney and bladder tissues, leukocytes and cultured normal urothelial cells revealed the mixture of nearly fully methylated and nearly unmethylated alleles that is typical of imprinted DMRs (Figure 4). In HepG2 cells, the sequence was homogeneously fully methylated in accord with the data of Anwar *et al*. [16]. In the two urothelial cancer tissues, differential methylation was still discernible. In contrast, the urothelial cancer cell lines presented a novel methylation pattern in which CpGs 1 to 6 and 10 to 16 were methylated, whereas CpGs 7 to 9 and 17 to 19 were unmethylated, thereby resulting in a ‘striped’ pattern (Figure 4).

Distinctive DNA methylation changes were also clearly evident in the *MEG3* DMR (Figure 4, right)*.* Benign kidney, leukocytes and benign bladder tissues predominantly harbored the typical pattern of DMRs with the expected mixture of either fully methylated or essentially unmethylated alleles (Figure 4). However, in urothelial cancer tissues and cell lines, a novel pattern was observed. Again, the methylation pattern of this region appeared striped insofar as CpGs 7 to 11 tended to be densely methylated and CpGs 1 to 4 sparsely methylated. In contrast, CpGs 5 and 6 were almost always unmethylated. Again, HepG2 cells were fully methylated, as expected.

We also analyzed several independent primary cultures of normal urothelial cells for methylation at the three sequences (Figure 4, [see Additional file 1: Figure S1A]). The methylation patterns at the *DLK1* promoter and the *MEG3* DMR in these cells were highly variable, tending towards a pattern intermediate between that of normal tissue and tumors. Differential methylation at the IG DMR appeared preserved.

### Pyrosequencing analysis confirms the methylation changes at the *MEG3*differentially methylated regions in urothelial cancer

**Figure 5:**
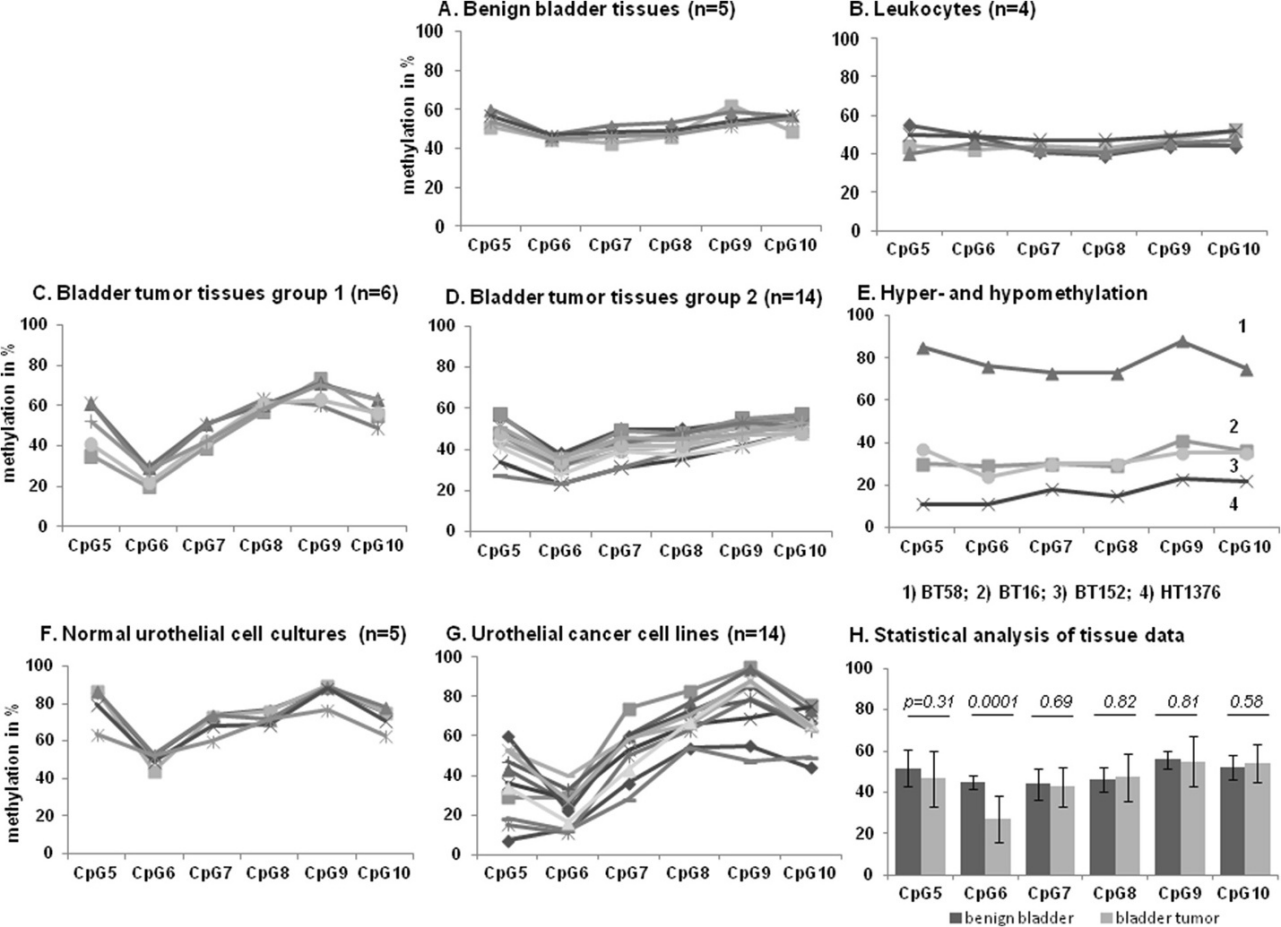
**Pyrosequencing analysis of DNA methylation at the** ***MEG3*** **differentially methylated region (DMR).** Methylation of the *MEG3 DMR* according to quantitative bisulfite pyrosequencing in **(A)** 5 benign bladder tissue samples, **(B)** 4 leukocyte samples, **(C-D)** 20 urothelial cancer tissues, **(E)** exceptional uniformly hypermethylated (BT58) and hypomethylated samples (cancer cell line HT1376, cancer tissues BT16, BT28). **(F, G)** Average methylation of the *MEG3* DMR in 5 normal urothelial cell cultures and 12 urothelial cancer cell lines. **(H)** Comparison of methylation at individual CpG positions in urothelial cancer tissues (light gray) compared to benign bladder tissues (dark gray); *P* values were obtained by student’s T-test. Please note that the number of lines in the graphs may appear lower than the respective ‘n’ due to overlays between samples with very similar values.

Since the most distinctive methylation changes occurred at the *MEG3* DMR we established a pyrosequencing assay interrogating CpGs 5 to 10 of this sequence to analyze a larger number of samples for the presence of the novel methylation pattern in a quantitative fashion. This assay was applied to a larger set of benign (n = 5) and tumor (n = 23) tissue samples, normal urothelial cells (n = 5) and urothelial carcinoma cell lines (n = 15) (Figure 5). In accord with the bisulfite sequencing results, all analyzed CpG positions were approximately 50% methylated in leukocytes and benign bladder tissues (Figure 5A and B). As predicted by bisulfite sequencing, methylation at CpG 6 was significantly decreased (*P* = 0.0001) in the majority of urothelial cancer tissues and cell lines (Figure 5H), whereas methylation at CpGs 7 to 10 was similar to normal tissues. Methylation at CpG 5 was more variable, but was often decreased in cancer tissues and cell lines (Figure 5C, D, G). In a few cancer tissues, methylation at CpG 9 was exceptionally high (Figure 5C). We furthermore observed a few cancerous samples with hypomethylation or hypermethylation at all analyzed CpGs compared to benign tissues (Figure 5E), including the cancer tissue BT152 also shown in Figures 3 and 4. For instance, the urothelial cancer cell line HT1376 retained approximately 20% methylation across all sites. Another example is the cancer tissue BT152, which appears to retain only a single copy of the locus (Figure 3), which according to the pyrosequencing and bisulfite sequencing assay (Figure 4) is heterogenously methylated. Cultured normal urothelial cells tended to display increased methylation across all CpG sites (Figure 5F), like UP124 in the bisulfite sequencing analysis shown in Figure 4.

These findings indicate the presence of a novel methylation pattern at the *MEG3* DMR in most urothelial tumor tissues and cell lines. In particular, reduced methylation at CpG 6 reliably distinguishes cancerous urothelial tissues and cell lines from the respective controls.

### COBRA analysis confirms normal and tumor methylation patterns at the *DLK1*promoter

The DNA sequence in the *DLK1* promoter is not well suited for methylation analysis by pyrosequencing. Therefore, to confirm that the consistent pattern of DNA methylation seen in urothelial cancer tissues and cell lines is not due to a cloning bias during bisulfite sequencing, we designed a COBRA assay. The region contains several TaqI restriction sites (TCGA), which are retained during bisulfite conversion if methylated, but mutated if unmethylated. Thus, up to five different restriction products are obtained if the sequence is heterogeneously methylated [see Additional file 1: Figure S2A]. Only two or three fragments are obtained, if the sequence is consistently methylated as suggested by the bisulfite sequencing results [see Additional file 1: Figure S2A]. Indeed, these expected patterns were obtained with DNA from exemplary urothelial cancer tissues and cell lines [see Additional file 1: Figure S2B].

Cumulatively, these findings indicate that the concomitant loss of *DLK1* and *MEG3* expression in urothelial cancer is associated with the acquisition of novel DNA methylation patterns, especially at the *DLK1* promoter and the *MEG3* DMR that in some cases extend to the IG DMR.

### Combined treatment with Aza-dC and SAHA induces *DLK1*and *MEG3*gene expression slightly

To determine to which extent DNA methylation contributes to the silencing of the two genes, we tested the effects of the DNA methyltransferase inhibitor 5-aza-2-deoxycytidine (Aza-dC) alone or in combination with the pan-HDAC inhibitor suberoylanilide hydroxamic acid (SAHA) on the expression of *DLK1* and *MEG3* mRNA in five urothelial cancer cell lines. Treatment with 5-aza-dC or SAHA individually did not significantly induce *MEG3* or *DLK1* expression except for 5-aza-dC in the BFTC905 cell line. Combined SAHA/Aza-dC treatment consistently restored *DLK1* and *MEG3* gene expression to detectable levels [see Additional file 1: Figure S3]. However, expression was still low suggesting that silencing of the two genes in urothelial cancer cells involves further mechanisms in addition to DNA methylation.

### Repressive histone modifications become strongly enriched at *DLK1-MEG3*regulatory elements in urothelial carcinoma

**Figure 6:**
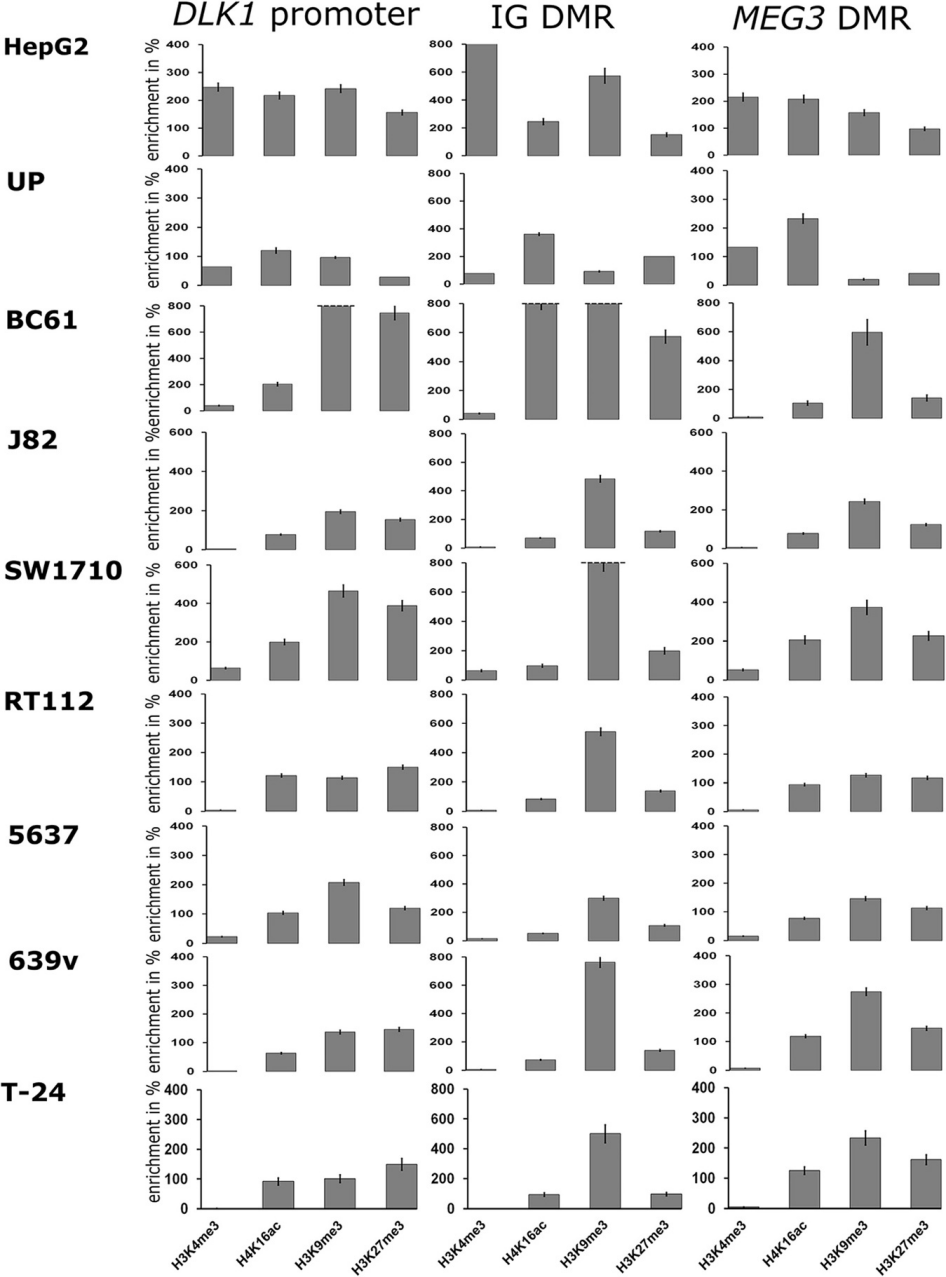
**Chromatin immunoprecipitation (ChIP) analyses of histone modifications at the** ***DLK1*** **promoter, the IG differentially methylated region (DMR) and the** ***MEG3*** **DMR.** Results of quantitative reverse transcription polymerase chain reaction (RT-PCR) conducted using DNA after ChIP with antibodies against active and repressive histone modifications *at the DLK1* promoter (left), IG DMR (center), *MEG3* DMR (right) in HepG2 and uncultured primary urothelial cells as controls and seven urothelial carcinoma cell lines derived from tumors of different stages and grades, that is, papillary urothelial cancer cell lines (BC61, J82, SW1710) and invasive urothelial cancer cell lines (5637, RT112, 639v and T24). *CTCFL* was used as reference for a silenced gene with repressive histone marks (H3K9me3 and H3K27me3), whereas *GAPDH* was used as control for a highly expressed gene with associated active histone modifications (H3K4me3 and H4K16ac). The 100% enrichment refers to the levels at the respective control genes. An alternative representation of the Figure and additional ChIP control experiments are displayed in Additional file 1: Figure S4.

Using chromatin immunoprecipitation (ChIP), we quantified the H3K4me3 histone modification associated with active genes, the H3K9me3 and H3K27me3 modifications associated with repression, and H4K16ac, a marker of transcriptional competence but also of fixed nucleosomes, at the *DLK1* promoter, the IG DMR and the *MEG3* DMR in normal urothelial cells, HepG2 cells and seven urothelial carcinoma cell lines (Figure 6 and [see Additional file 1: Figure S4]).

Because the variable results of DNA methylation analyses had suggested that the epigenetic state of the 14q32 region changes during culture of normal urothelial cells (Figure 4, [see Additional file 1: Figure S1]), we used freshly prepared, uncultured urothelial cells for the analysis of histone modifications (Figure 6). As expected for an imprinted bivalent domain, these normal urothelial cells displayed enrichment of H3K4me3 and H3K9me3 at comparable levels and slightly more enriched H4K16ac at the *DLK1* promoter. The IG DMR likewise displayed enrichment of both active and repressive histone modifications, H3K4me3 and H3K9me3, and more strongly H3K27me3. Interestingly, H4K16ac was strongly enriched at both DMRs. At the *MEG3* DMR, in agreement with the high expression in normal bladder, the active histone modification H3K4me3 was enriched, whereas repressive histone modifications (H3K9me3 and H3K27me3) were low.

HepG2 cells were used as a *DLK1* expressing control cell line. In HepG2 cells, active and repressive histone modifications were enriched to comparable extents at the *DLK1* promoter and the *MEG3* DMR. The active mark H3K4me3 was highly enriched at the IG DMR in HepG2 cells, with higher levels of H3K9me3 compared to H3K27me3, which is the inverse pattern compared to that in normal urothelial cells.

The most striking difference in the urothelial carcinoma cell lines towards the controls was the severe depletion of H3K4me3 at all three regulatory regions analyzed. In comparison, repressive histone modifications (H3K9me3 and H3K27me3) were generally increased, in particular H3K9me3 at both DMRs, whereas increases in H3K27me3 were more evident at the *DLK1* promoter and the *MEG3* DMR. As a consequence, these latter two regions assumed similar patterns of histone modifications across the urothelial carcinoma cell lines. Interestingly, in contrast to the H3K4me3 mark, the H4K14ac modification was less severely depleted or even retained in some cell lines, with lowered levels especially at the IG DMR.

In summary, the ChIP analyses revealed the predominance of repressive histone modifications and a nearly complete loss of H3K4me3, with partial retention of H4K16ac, a modification characteristic of fixed nucleosomes, across the entire analyzed region in all urothelial carcinoma cells. This finding supports the contention that the region acquires a repressive chromatin state in urothelial carcinoma.

## Discussion

Previous molecular and cytogenetic analyses of urothelial cancers have suggested at least one tumor suppressor gene residing at chromosome 14q32.2 [25, 27, 32]. This chromosomal region contains a cluster of imprinted genes important for early embryonic mammalian development. The genes *DLK1* and *MEG3* within this cluster are tumor suppressor candidates in urothelial carcinoma due to their known functions in the regulation of cell growth and development [20, 33]. Indeed, *DLK1* is downregulated by epigenetic mechanisms in renal cell carcinoma [5]. However, it is upregulated in hepatocellular carcinoma cell lines and tissues, acute myeloid leukemia and adrenocortical tumors, as well as in breast, ovarian and cervical cancer cell lines, suggesting tissue-specific functions [5, 14, 30, 34]. The long noncoding RNA *MEG3* was reported to act as a tumor suppressor, too, but in a more consistent manner [1, 18, 19, 22]. In those studies on other cancers where DNA methylation had been investigated, homogeneous methylation patterns were reported in the investigated regions resulting from allelic loss or consistent with epitype switching [16]. Of note, no previous study has characterized the epigenetic state of the 14q32.2 cluster beyond DNA methylation. In the present study, we used the hepatocellular carcinoma line HepG2 as a control and obtained results consistent with previous findings [5, 16, 30]. In additional experiments (data not shown), we also confirmed the reported changes in a renal carcinoma cell line [5, 30]. With respect to HepG2, the homogeneous DNA methylation patterns, the copy number measurement and the sole expression of *DLK1* argue strongly that this cell line retains only a paternal allele.

In previous reports on other cancer types, either *DLK1* or *MEG3* was reported to become deregulated, but not both genes, as we observed in urothelial carcinoma. Unfortunately, many papers do not comment on whether they have investigated the other gene at all. In benign bladder tissues, *DLK1* and *MEG3* were well detectable with *MEG3* being expressed more strongly than *DLK1*, like in normal kidney, liver and pituitary gland [5, 30, 35]. Various models for the inverse regulation of the genes in the 14q32 imprinting cluster have been suggested [8]. Most models assume that the IG DMR activates transcription of maternally expressed RNA genes such as *MEG3* and is required for silencing of the paternally expressed genes such as *DLK1*. Despite their reciprocal relationship in benign bladder tissue, *MEG3* and *DLK1* expression were found to be both significantly diminished in urothelial cancer tissues and cell lines. With respect to *MEG3*, our findings are fully consistent with those of Ying *et al*. [29]. Unfortunately, neither these authors nor others have explicitly reported on *DLK1* in urothelial carcinoma.

Importantly, *DLK1* is expressed from the paternal allele and *MEG3* from the maternal allele. Their concomitant downregulation is therefore difficult to explain by allelic loss. Accordingly, we found a range of copy numbers between one and four in urothelial carcinoma tissues and cell lines indicating that both losses and gains of this region are frequent, accounting for the high frequency of apparent ‘loss of heterozygosity’ in previous reports [23, 26]. However, downregulation of the two genes was observed irrespective of whether copy numbers increased, decreased or remained steady. In particular, the copy numbers of the three sequences assayed in the region remained identical within each sample, excluding partial changes. Therefore, concomitant downregulation of both *DLK1* and *MEG3* can indeed not be explained by chromosomal deletions.

By a similar argument, we can exclude conventional loss of imprinting or epitype switching as a plausible cause of the concomitant downregulation. If the gene cluster assumed the maternal state, *MEG3* expression should be retained or even increased, and conversely, if the gene cluster assumed the paternal state, *DLK1* expression should be retained or increased. Likewise, DNA methylation at the regulatory regions should become homogeneous and resemble either the maternal or the paternal pattern. This type of change is exemplified by the HepG2 cell line, which shows strong *DLK1* expression associated with a paternal epigenetic state.

A clue to the actual mechanism is provided by the observation that the DNA methylation patterns at the three regulatory regions in urothelial carcinoma cells are indeed homogeneous, but are different from both the maternal and paternal patterns in normal bladder and renal tissues. These methylation patterns therefore suggest that a novel repressed epigenetic state is established during urothelial carcinogenesis at the 14q32 gene cluster. Of note, several microRNAs encoded in the *DLK1*-*MEG3* cluster (Figure 1) within *RTL1* (for example, miR127, miR136, miR431 and miR433) and between *RTL1* and *DIO3* (miR376a, miR487b, miR382, miR380-5p and miR412) have also been described to be significantly reduced or silenced by DNA hypermethylation in bladder tumor tissues and the cell lines RT4, RT112 and T24 [19, 29] suggesting that silencing may extend across a large part or the entire imprinted gene cluster at 14q32. Interestingly, a coordinated regional epigenetic change has been reported for another, more centromeric, region at chromosome14q12 in urothelial carcinoma [16].

The repressed state of the *DLK1*-*MEG3* cluster in urothelial carcinoma cell lines is also reflected in the predominance of repressive histone modifications such as H3K9 and H3K27 trimethylation (Figure 6). Interestingly, whereas other active modifications were lost, H4K16ac was largely retained, despite the observation that mono-acetylated H4K16 tends to become lost in cancer in general [36–38]. This modification often indicates transcriptional competence, but it is also associated with fixed nucleosomes [36–38]. We speculate that the repression of the cluster may be accompanied by rigid nucleosomal positioning interacting with DNA methylation as documented in other cases [39]. This hypothesis is further supported by mathematical models of how DNA methylation at CpG sites changes the physical properties, positioning and phasing of nucleosomes [40, 41]. Indeed, an overlay of nucleosome prediction at all analyzed regulatory regions (*DLK1* promoter, *MEG3* and IG DMR) with our experimentally obtained methylation patterns suggest strongly positioned and potentially newly phased nucleosomes in cancer compared to benign urothelial cells (Additional file 1: Figure S5). In particular, altered nucleosomal positioning could account for the peculiar patterning of DNA methylation at the *MEG3* DMR, where one specific CpG site (#6) became significantly hypomethylated in cancer cells, while methylation of flanking sites rather increased. It could therefore be interesting to map the nucleosomal positioning in the 14q imprinted gene cluster in normal and cancer cells in future work.

Our study of the epigenetic changes at the *DLK1*-*MEG3* cluster in urothelial carcinoma was hampered by the lack of an epigenetically stable normal urothelial cell line. Upon culturing, normal urothelial cells acquire a considerable degree of plasticity, including the ability to differentiate into epidermis-like as well as urothelial-like structures [42]. Upon immortalization by telomerase expression, further changes ensue, in particular, deregulation of key epigenetic regulators [43]. The *DLK1*-*MEG3* cluster appears particularly susceptible to such changes, as reduced expression or silencing of *MEG3* has also been observed in normal cell lines originating from other tissues [35, 44]. Changes in the expression of imprinted genes, specifically of *Meg3*, have also been reported during establishment of cell cultures of mouse embryonic fibroblasts [45]. In our study, this epigenetic instability manifested as partial changes in DNA methylation at *DLK1* and *MEG3* that varied between individual urothelial cell cultures. These changes did not extend to the IG DMR and were not as pronounced as in the cancer cell lines and tissues. For that reason, we used freshly isolated, noncultured urothelial cells, which are unfortunately only available in limited amounts, for the chromatin immunoprecipitation experiments.

## Conclusions

In conclusion, our data suggest that the 14q32 imprinted gene cluster acquires a novel epigenetic state in urothelial cancer that allows the concomitant inactivation of *DLK1* and *MEG3* expression, overcoming the normally antagonistic regulation of these two imprinted genes. One target of these changes is evidently *MEG3,* which emerges as a tumor suppressor in many different tissues [46]. In urothelial carcinoma, specifically, Ying *et al*. [29] have demonstrated its tumor suppressor activity and our study confirms the remarkably high frequency of its downregulation reported by these authors. However, the inactivation of *MEG3* alone could be achieved by conventional mechanisms such as allelic loss or by epitype switching. The findings reported here and the observation of others that several smaller RNA species encoded in the cluster are downregulated by DNA hypermethylation [19, 29], collectively suggest that in urothelial cancers, a regional silencing process additionally targets other genes, including potentially *DLK1*. The question of which of these changes support tumor progression will therefore have to be addressed by future research.

## Methods

### Tissue samples

The bladder cancer and benign tissue samples were a subset of those described in previous studies [2, 47] comprising 11 benign bladder tissues (morphologically normal tissue from tumor cystectomies) and 30 bladder cancer tissues from 25 male and 5 female patients ages from 54 to 84 years (median age: 66 years). The tumor stages and grades according to the current UICC classification were as follows: pT3 G3 in 11 cases, pT4 G3 in 6 cases, pT2 G2 in 6 cases, pT2 G3 in 3 cases and one case each of pT3 G2, pT1 G2, pTa G3 and pTa G2. The study was approved by the ethics committee of the medical faculty of the Heinrich Heine University, and all patients gave written consent to the use of their tissues.

### Cell lines and cell culture

All urothelial cancer cell lines (5637, 639v, 647v, BFTC905, HT1376, J82, RT4, RT112, SD, SW1710, Umuc3, VmCub1, T24) and the hepatocellular carcinoma cell line HepG2 were cultured in DMEM (Gibco, Darmstadt, Germany) supplemented with 10% fetal calf serum [48]. They were obtained from the DSMZ (Braunschweig, Germany), except for Umuc3 [49], kindly provided by Dr. Grossman, Houston. The well-differentiated urothelial carcinoma cell line BC61 derived from a papillary bladder cancer in our lab was cultured as previously described [49, 50]. Primary urothelial cells (UP) were prepared from ureters after nephrectomy and were routinely maintained in keratinocyte serum-free medium (KSFM, Gibco, Darmstadt, Germany) supplemented with 12.5 μg/ml bovine pituitary extract and 0.25 ng/ml epidermal growth factor as described [50].

### RNA isolation and quantitative reverse transcription polymerase chain reaction

Total RNA was isolated from subconfluent cell cultures and cell lines or from powdered tissues using the RNeasy Mini or Micro Kit (Qiagen, Hilden, Germany). Two μg RNA was reverse transcribed using 200 U SuperScriptII reverse transcriptase (Invitrogen, Darmstadt, Germany), with 300 ng oligo-dT and 25 ng random hexamer primers in a reaction volume of 20 μl. Real-time PCR assays were performed with the ABI7900HT System using the QuantiTect SYBR Green PCR Kit (Qiagen, Hilden, Germany) and QuantiTect primer assays for *DLK1* and *TBP* in a reaction volume of 25 μl. *MEG3* primers were described by Kawakami *et al*. 2006 [5]. *TBP* was used as a reference gene. Primers and QuantiTect assays are listed in Additional file 1: Table S1. Statistical comparisons between benign bladder and tumor bladder tissue expression were made by the Mann-Whitney U Test with SPSS 21.

### DNA isolation and bisulfite sequencing

Total genomic DNA was isolated from subconfluent cell cultures using the Blood and Cell Culture DNA Midi Kit (Qiagen, Hilden, Germany). For DNA methylation analyses, 1 μg DNA was bisulfite converted using the EZ DNA Methylation-Gold Kit (Zymo Research, Freiburg, Germany) following the supplier’s protocol with incubations at 95°C for 10 min, followed by 64°C for 2.5 h. After purification, the bisulfite converted DNA was analyzed for methylation of the *DLK1* promoter and the *MEG3* DMR using primers described in [5] and for the IG DMR as described in [13]. PCRs were performed in a Biometra Thermocycler using an annealing temperature of 55°C. The reaction mix was composed of 40 ng bisulfite converted DNA, 150 μM dNTP, 15 pmol primer each and 1.25 U HotStar Taq Polymerase (Qiagen, Hilden, Germany) in the appropriate buffer. PCR products were cloned into pCR4-TOPO. Plasmid DNA was isolated using the Fast Plasmid Mini Kit (5Prime, Hamburg, Germany) and several clones were sequenced using standard methods by our BMFZ core facility.

### DNA methylation analysis by pyrosequencing

For pyrosequencing analysis of the *MEG3 DMR* bisulfite-treated DNA samples were used with newly designed primers [see Additional file 1: Table S1] using the following conditions: initial denaturation step at 95°C for 15 min, followed by 42 cycles consisting of denaturation at 95°C for 20 s, annealing at 57°C and extension at 72°C for 30 s. Pyrosequencing was carried out on a PyroMark Q24 instrument (Qiagen) according to the manufacturer's instruction. This pyrosequencing analysis interrogated the CpG positions 5 to 10 in the *MEG3 DMR* region analyzed by standard bisulfite sequencing.

### Gene copy number analysis

For gene copy number analysis by qPCR 25 ng total genomic DNA was used with the QuantiTect SYBR Green PCR Kit (Qiagen, Hilden, Germany) and primers for *DLK1*, IG DMR and *MEG3* DMR as in ChIP analysis [see Additional file 1: Table S1]. PCR conditions were as follows: 95°C for 15 min, followed by 30 cycles of 94°C for 15 s, annealing (55 to 60°C) for 30 s, 72°C for 10 to 14 s. *GAPDH* was used as a reference gene. Gene copy numbers were calculated as the ratio of the measured sequence to the reference gene *GAPDH* and normalized to the value from leukocyte DNA set as two copies.

### Chromatin immunoprecipitation

Chromatin immunoprecipitation (ChIP) was performed on triplicate samples with the ChIP-IT Express Kit (Active Motif, Rixensart, Belgium) according to the manufacturer’s instructions as previously described [51] or 10^5^ primary urothelial cells freshly prepared from a ureter after nephrectomy using the True MicroChIP kit (Diagenode, Liege, Belgium). The precipitated DNA fractions were quantified by real-time PCR with the QuantiTect SYBR Green PCR Kit (Qiagen, Hilden, Germany) and primers for the *DLK1* promoter, IG DMR and *MEG3* DMR. ChIP assays [see Additional file 1: Table S1] were located in the regions analyzed for DNA methylation. PCR conditions were as follows: 95°C for 15 min, followed by 45 cycles of 94°C for 15 s, annealing (55 to 60°C) for 30 s, 72°C for 30 s. Isotype (IgG) control values were subtracted. *CTCFL* was used as a control for a silenced gene with inactive histone modifications (H3K9me3 and H3K27me3), whereas *GAPDH* was used as control for a highly expressed gene with active histone modifications (H3K4me3, H4K14ac). The input DNA purified from the sheared unprecipitated chromatin was used to make a standard curve for each gene investigated and each sample was measured in duplicate with less than 12% variation. The 100% enrichment refers to the levels at the respective control genes. The results remained in principle unchanged, if the values were normalized to input DNA directly (compare Figure 6 and [Additional file 1: Figure S4A]). Also, the enrichment of the H3K27me3 modification compared to the H3K4me3 modification at the *DLK1* promoter, IG DMR and *MEG3* DMR of UC cell lines remained clearly evident when, in an independently performed experiment, the values were normalized to input DNA as well as immunoprecipitated total H3 [Additional file 1: Figure S4B]. The following antibodies were used: anti-H3K4me3, anti-H3K27me3 (Abcam, Cambridge, UK), anti-H3K27me3, anti-H4K16Ac, anti-H3 (Active motif, La Hulpe, Belgium).

## Electronic supplementary material

Additional file 1: Table S1: Primer assays. **Table S2.** Copy number changes and relative to gene expression of *DLK1* in urothelial cancer cell lines. **Figure S1.** Additional bisulfite sequencing results in urothelial cultures. **Method S1.** Treatment with epigenetic inhibitors. **Figure S2.** COBRA analysis of the *DLK1* promoter sequence. **Method S2.** DNA methylation analysis by COBRA. **Figure S3.** Effects of treatment with epigenetic inhibitors on *DLK1* and *MEG3* expression. **Figure S4.** Comparison between two normalization methods for the ChIP experiment. **Figure S5.** Bioinformatic prediction of nucleosome positioning at the *DLK1-MEG3* locus. (DOCX 1 MB)

## Abbreviations

ChIP: chromatin immunoprecipitation
DMR: differentially methylated region
ICR: imprinting control region
IG: intergenic
UCC: urothelial cancer cell
UP: (normal) urothelial cell culture.
