# Supplementary material for: Concomitant downregulation of the imprinted genes DLK1 and MEG3 at 14q32.2 by epigenetic mechanisms in urothelial carcinoma
Source: Clin Epigenetics. 2014 Nov 23;6(1):29. doi: 10.1186/1868-7083-6-29 (PMC4348104; doi:10.1186/1868-7083-6-29)
Supplement: Supplementary file 1 — Additional file 1: Table S1: Primer assays. Table S2. Copy number changes and relative to gene expression of DLK1 in urothelial cancer cell lines. Figure S1. Additional bisulfite sequencing results in urothelial cultures. Method S1. Treatment with epigenetic inhibitors. Figure S2. COBRA analysis of the DLK1 promoter sequence. Method S2. DNA methylation analysis by COBRA. Figure S3. Effects of treatment with epigenetic inhibitors on DLK1 and MEG3 expression. Figure S4. Comparison between two normalization methods for the ChIP experiment. Figure S5. Bioinformatic prediction of nucleosome positioning at the DLK1-MEG3 locus. (DOCX 1 MB) [file 13148_2014_102_MOESM1_ESM.docx]

**Additional file 1**

**Table S1** *Primer used for gene expression, bisulfite sequencing, pyrosequencing, COBRA, NuSA, quantitative DNA- and ChIP-Real Time PCR analysis.*

| Gene | Primer | Sequence 5’-3’ | °C | bp |
| --- | --- | --- | --- | --- |
| DLK1 | DLK1_QT_Assay | QT00093128 | 55 | 136 |
|  | BiSeq_DLK1PromR2-F | GTTTTTATGGTTAGGGGTATAGGG | 59.3 | 204 |
|  | BiSeq_DLK1PromR2-R | CAAACCTCCCCRAAAAACACAATT | 58.4 |  |
|  | MS-DLK1-TaqI-F | GTATATATAGGTTTGTTTAGGATA | 52 | 280 |
|  | MS-DLK1-TaqI-R | TACCACCAAACAACTACATTTTT | 53 |  |
|  | ChIP_DLK1 Fwd | AGTGTTTCGGTGTTCCTG | 53.7 | 88 |
|  | ChIP_DLK1 R1 | GCCTGCCTAGGACAAGTC | 58.2 |  |
|  | ChIP_DLK1 R2 | TACACGTTCCCTCACACTG | 56.7 | 136 |
| MEG3 | qPCR_MEG3 F | CTGTCTACACTTGCTGTCTT | 58 | 259 |
|  | qPCR_MEG3 R | TTCCCACGTAGGCATCCAGG | 64 |  |
|  | BiSeq_DMR-F | GTAAGTTTTATAGGTTGTAAAGGGGGT | 62.7 | 216 |
|  | BiSeq_DMR-R | CCACAACTAATAACTAAAAAAATAAACATT | 55.8 |  |
|  | ChIP_DMR F1 | GCTGTTTCCTAGCTATTAATACTG | 57.6 | 82 |
|  | ChIP_DMR R1 | CGCTTCAAAAAATCATGG | 49.1 |  |
|  | ChIP_DMR F2 | GTGTCTACGACAGCCTCC | 58.2 | 233 |
|  | ChIP_DMR R2 | CTAGGAAACAGCCAGAGC | 56.0 |  |
|  | PyrSeq_MEG3_FBiotin | AGTTAATGATTAGGGAGGTGAATATTGAT | 60 | 241 |
|  | PyrSeq_MEG3_R | TCCCAAACTCTAATCCCTAAAACTCCT | 62 |  |
|  | MEG3DMR-Seq | TCTCTATCTCCCCAACAATA | 53 |  |
| IG DMR | BiSeq_IGDMR-F | GTTAAGAGTTTGTGGATTTGTGAGAAA | 58.9 | 429 |
|  | BiSeq_IGDMR-R | CTAAAAATCACCAAAACCCATAAAATC | 57.4 |  |
|  | ChIP_IGDMR_M_F1 | CTTGCTAATTGCCAGCGAT | 54.5 | 73 |
|  | ChIP_IGDMR_M_R1 | GGATTACGGGTTTAGCGGA | 56 |  |
|  | ChIP_IGDMR_U_F2 | CAATGGACTCGCCCTTTAG | 56.7 | 96 |
|  | ChIP_IGDMR_U_R2 | AACCATGGCGAATTGTGGT | 54.5 |  |
|  | NuSA_ICR-1F38 | GTCCCCAAGTAGAGGGTG | 58 | 67 |
|  | NuSA_ICR-1R105 | TCCAGCCTGGGGTGTAAAC | 58 |  |
|  | NuSA_ICR-2F308 | GTTGCCCATGGCTTGCTAAT | 57 | 47 |
|  | NuSA_ICR-2R355 | ACCACTCGCAATTGGCAAAT | 55 |  |
|  | NuSA_ICR-6F94 | CCCCAGGCTGGAATTGCTAAG | 61 | 81 |
|  | NuSA_ICR-6R175 | CACAGGCAACTGGCAAGGCC | 63 |  |
|  | NuSA_ICR-8F387 | ATCCTGTGGTACTGTAACTG | 55 | 73 |
|  | NuSA_ICR-8R460 | CTGCATTTGGGCAAAAGAGG | 57 |  |
|  | NuSA_ICR-9F443 | TCTTTTGCCCAAATGCAGTTCT | 56 | 91 |
|  | NuSA_ICR-9R534 | AGAATCACCAAGACCCATGAA | 56 |  |
|  | NuSA_ICR-12F551 | TACGGTCCTCAGGCAACTTC | 60 | 83 |
|  | NuSA_ICR-12R634 | GGGGAGGTGCAGGACACAA | 61 |  |
| reference genes | TBP_QT_Assay | QT00000721 | 55 | 132 |
|  | NuSA_TBP_F | CTGTTCCACCAAGAAAGTT | 52 | 122 |
|  | NuSA_TBP_R | ATAAGGCATCATTGGACTAAA | 52 |  |
|  | ChIP_GAPDH_F | TACTAGCGGTTTTACGGGCG | 60 | 166 |
|  | ChIP_GAPDH_R | TCGAACAGGAGGAGCAGAGAGCGA | 66 |  |
|  | NuSA_GAPDH_F94 | GGCGGCCTCCGCATTGCA | 62 | 70 |
|  | NuSA_GAPDH_R164 | CCCACCAGGCCTCCATGC | 62 |  |
|  | ChIP_CTCFL_F | GAACAGCCCATGCTCTTGGAG | 62 | 113 |
|  | ChIP_CTCFL_R | CAGAGCCCACAAGCCAAAGAC | 62 |  |

**Table S2:** C*opy number changes and relative gene expression of DLK1 in urothelial cancer cell lines.*

*The copy number in normal leukocytes is set as 2. The expression changes observed for DLK1 occur independent of copy number changes.*

| **cell line** | **Copy number*** | **Expression** |
| --- | --- | --- |
| BC61 | 2 | 0 |
| SW1710 | 0.5 | 0.01 |
| UM-Uc3 | 3 | 0.12 |
| VmCub1 | 3 | 0 |
| J82 | 2 | 0 |
| 253J | 0.5 | 0 |
| 5637 | 2 | 0 |
| 639v | 3 | 0 |
| 647v | 2 | 0 |
| BFTC905 | 2 | 0.05 |
| HT1376 | 4 | 0.07 |
| RT4 | 2 | 0.06 |
| RT112 | 4 | 0 |
| SD | 4 | 0 |
| T-24 | 2 | 0 |

**Figures**

**Figure S1**

**Figure S1** *Additional bisulfite sequencing results in normal urothelial cells*

Bisulfite sequencing results of 11 CpGs each in the *DLK1* promoter region and the *MEG3* DMR in two urothelial cell cultures clearly show a highly variable methylation pattern during normal proliferating urothelial cell cultures.

**Figure S2**


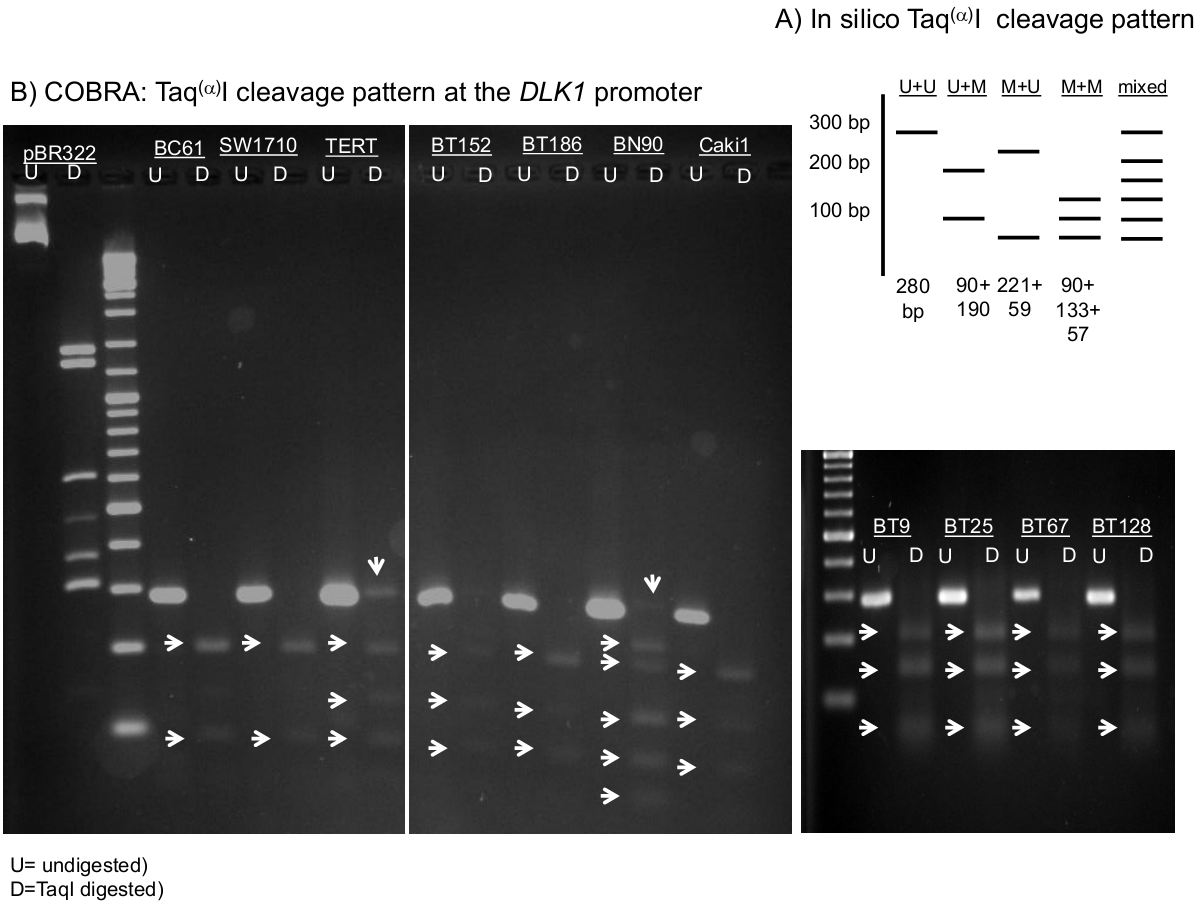


**Figure S2** *COBRA analysis of the DLK1 promoter sequence.*

**A)** in-silico predicted TaqI cleavage pattern depending on the methylation state (U = unmethylated, M = methylated) of the two TaqI sites **(B)** TaqI cleavage pattern at the *DLK1* promoter in urothelial cancer (BC61, SW1710) and normal cell lines (TERT), normal (BN) and cancerous (BT) bladder tissues and Caki1 cells. Variable methylation seen in benign tissues should lead to a mixture of all possible products (as illustrated in normal bladder BN90). In the striped methylation pattern found by bisulfite sequencing one TaqI site is predicted to reside in a methylated sequence and one TaqI site is in the unmethylated sequence - this pattern is most clearly evident in the two bladder cancer cell lines BC61 and SW1710. Bladder tumor tissues, immortalized normal urothelial cells and Caki1 cells show an additional band indicating methylation of both sites in some alleles.

**Figure S3**

**A)**

**B)**

**Figure S3** *Effects of treatment with epigenetic inhibitors on DLK1 and MEG3 expression****.* A)** Urothelial cancer cell lines VmCub1, J82, 5637, SW1710 and BFTC905 were treated with aza-dC (5-aza-2-deoxycytidine) or SAHA (suberoylanilide hydroxamic acid) for three days. Neither Aza-dC nor SAHA treatment significantly induced *MEG3* expression, except for BFTC905. **B)** Urothelial cancer cell lines VmCub1, J82, 5637, SW1710 and BFTC905 were treated with aza-dC (5-aza-2-deoxycytidine) and SAHA (suberoylanilide hydroxamic acid) for three days. Expression of *DLK1* and *MEG3* was undetectable by qRT-PCR in untreated control cells, dark grey bars indicate *DLK1* expression and light grey bars *MEG3* expression in treated cells. (-) represents untreated cells, (+) indicates SAHA plus aza-dC treated cells

**Figure S4**

1. Normalization to input DNA
2. Normalization to input DNA and Histone H3 enrichment

**Figure S4 C***omparison between two normalization methods for the ChIP experiment*

**A)** Reanalyzed ChIP results from Figure 6. Each diagram represents one locus and contains the relative enrichment of active (green) and repressive (red) histone modifications, as well as control IgG (grey) normalized to input DNA per sample. GAPDH and CTCFL are control loci for actively transcribed and repressed genes in urothelial cancer, respectively. Urothelial cancer cell lines are T-24, 639v, 5637, J82, BC61 and SW1710; UP is one normal proliferative urothelial cell culture. **B)** Effect of normalization on histone H3 and input. In an independent experiment H3K4me3 and H3K27me3 enrichment was meausred for 5 urothelial carcinoma cell lines and normalized to total H3 determined in the same experiment. Each diagram represents one locus and contains the relative enrichment of one active (green) and one repressive (red) histone modification for a selected set of samples normalized to input DNA and the relative enrichment of Histone H3. Note that the pattern of modifications at the DLK1 promoter and the IG and MEG3 DMRs resembles that of the inactive locus CTCFL. This is fully consistent with the results shown in panel A and Fig. 6 in the main text.

**Figure S5**


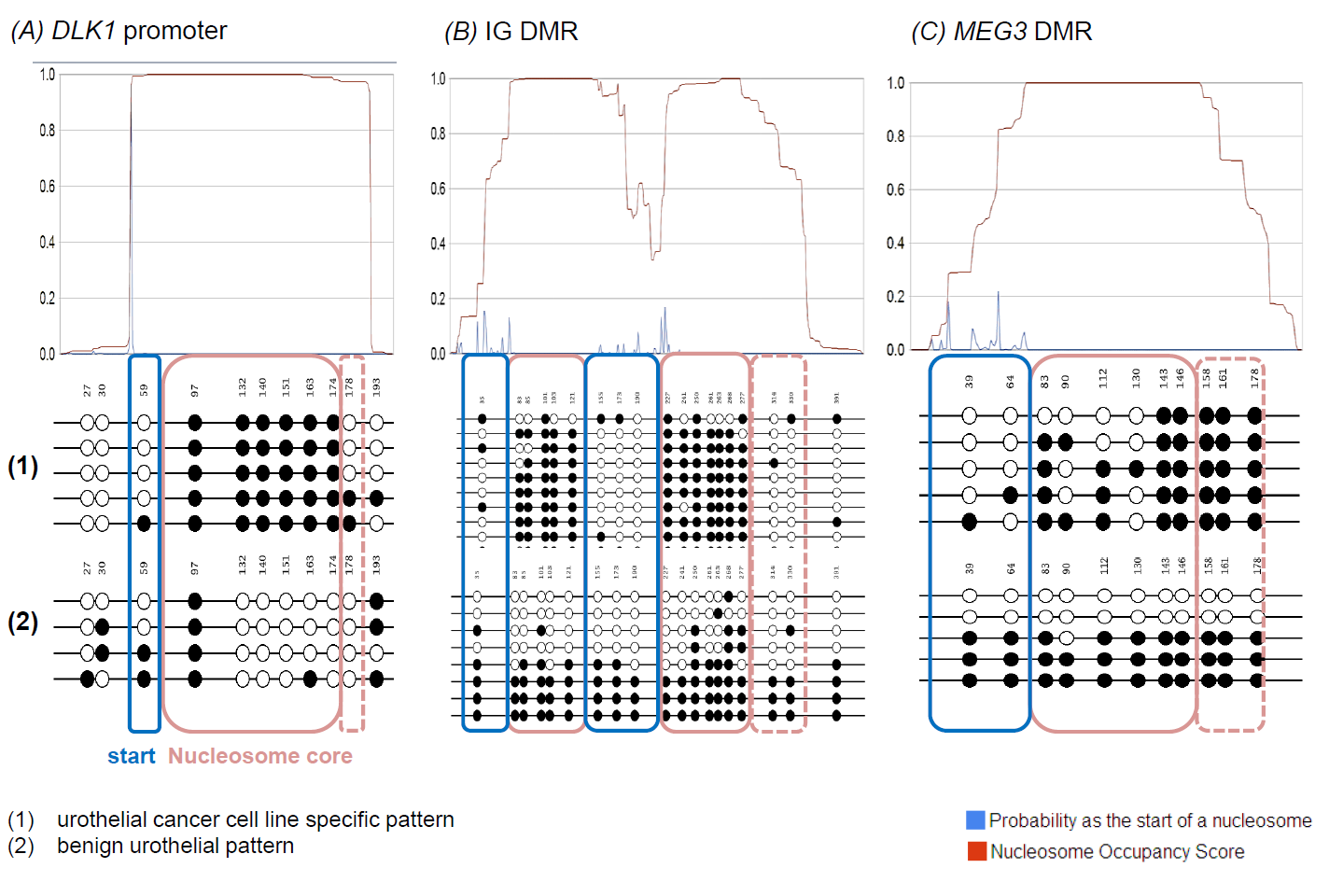


**CpG6**

**Figure S5** *Bioinformatic prediction of nucleosome positioning at the DLK1-MEG3 locus*

*In silico* analysis of nucleosome positioning by NuPOP prediction [[1](#_ENREF_1)] in (**A**) *DLK1* promoter, (**B**) IG DMR and (**C**) *MEG3 DMR* regulatory regions, compared to typical bisulfite sequencing results in benign tissues and urothelial cancer cell lines. The start of a nucleosome is highlighted in blue, the nucleosome core is marked in red. An overlay of the predicted nucleosomes in the DLK1, IG DMR and MEG3 DMRs with the observed methylation patterns clearly suggest an increased methylation of the nucleosome core and reduced methylation of the linker maybe resulting in the striped methylation pattern. In the *MEG3* DMR the significantly demethylated CpG6 position is predicted to be in a nucleosome core and furthermore there is no evidence for binding of transcription factors as assessed by the free web tool TFSEARCH [[2](#_ENREF_2)].

**Methods**

**Treatment with epigenetic inhibitors.**

For inhibitor treatment, 5-aza-2-deoxycytidine (Sigma Aldrich, Munich, Germany) was added at a final concentration of 2 µM (5 µM for Caki-1) in fresh medium daily for three days. For combination experiments from day 2, 2 µM suberoylanilide hydroxamic acid (Cayman Chemical Company, Tallinn, Estonia) was added.

**DNA methylation analysis by** **COBRA**.

For *Combined Bisulfite Restriction Analysis* (COBRA) of the *DLK1* promoter bisulfite-treated DNA samples were amplified with primers MS_DLK1_Taq (Additional file 1: table1) to yield a 280 bp product, which is encompassed by the sequence analyzed by bisulfite sequencing. After column purification, the PCR product was digested using 20 U *TaqI* at 65°C for 1 h and the digestion products were separated on a 3% agarose gel.

**Additional file 1: References**

[1] Xi L, Fondufe-Mittendorf Y, Xia L, Flatow J, Widom J, et al. (2010) Predicting nucleosome positioning using a duration Hidden Markov Model. BMC bioinformatics 11: 346.

[2] Heinemeyer T, Wingender E, Reuter I, Hermjakob H, Kel AE, et al. (1998) Databases on transcriptional regulation: TRANSFAC, TRRD and COMPEL. Nucleic Acids Res 26: 362-367.
